# Gut Microbiota–Brain Axis in Regulation of Feeding Behavior

**DOI:** 10.3390/microorganisms11030749

**Published:** 2023-03-14

**Authors:** Sergueï O. Fetissov

**Affiliations:** Regulatory Peptides-Energy Metabolism and Motivated Behavior Team, Neuroendocrine, Endocrine and Germinal Differentiation and Communication Laboratory, Inserm UMR1239, University of Rouen Normandie, 76000 Rouen, France; serguei.fetissov@univ-rouen.fr

**Keywords:** gut microbiota, dysbiosis, probiotics, autoprobiotics, feeding behavior, appetite, obesity, metabolic diseases, anorexia, neuropsychiatric diseases

## Abstract

The survival of microorganisms inhabiting the intestinal tract depends on the nutrients provided by the host, with the latter obtaining them through food intake. It is hence not surprising that the co-evolution of gut bacteria and their hosts, including humans, shaped intrinsic interactions between their respective metabolisms with an impact on host feeding behavior. Understanding molecular pathways underlying such interactions may aid in the development of new therapeutic approaches for several pathological conditions accompanied by altered feeding behavior. A Special Issue titled “Gut Microbiota–Brain Axis in Regulation of Feeding Behavior” contributes to this topic of research, with eight papers covering its various aspects such as autoprobiotics, metabolic diseases and anorexia.

## 1. Introduction

The topic of research involving gut microbiota in the regulation of feeding behavior is rapidly evolving. Indeed, since the initial 2017 paper written by this editor which conceptualized the alternation of host appetite cycles as a result of an intrinsic link between microbial and host metabolism [1], several studies contributed to this field and confirmed the role of gut bacteria in the regulation of host feeding behavior [2,3,4]. Nevertheless, this Editorial is not an attempt to review such contributions, but to present an overview of a Special Issue titled “Gut Microbiota–Brain Axis in Regulation of Feeding Behavior” recently published in this journal. This Special Issue includes eight papers, each covering different aspects of this research topic. The research areas covered by these papers can be grouped into the role of gut microbiota in pathological conditions such metabolic diseases and anorexia.

A new therapeutic approach of using the indigenous gut bacteria as autoprobiotics has gained attention due to its advantage of increased tolerability at the individual level. As such, in the study by Ermolenko et al., the authors use autoprobiotics in patients with metabolic syndrome and obtain a decrease in body mass index (BMI) [5]. The treatment did not, however, modify feeding behavior, suggesting that the molecular mechanisms underlying the BMI improvement were independent of the regulation of appetite. This can be linked to an increase in some Lactobacilli strains with probiotic supplementation as was previously shown to slightly improve obesity but not hyperphagia [6]. One of the main indications for taking autoprobiotics is recovery from gut dysbiosis induced by antibiotic treatment. The potential effect of autoprobiotics on the feeding behavior of rats in such circumstances was evaluated by Gromova et al. [7]. The authors noticed a decrease in food intake after antibiotic treatment, which is a typical response. After antibiotic removal, both the rats receiving and not receiving Bifidobacteria-based autoprobiotics increased their food intake. Thus, autoprobiotics improved dyspeptic symptoms without influencing feeding behavior, in agreement with the above-cited study in humans with metabolic syndrome.

The research of probiotic selection for the treatment of metabolic syndrome and obesity should consider the sex differences. In fact, the study by Myles et al. revealed that *Lactobacillus helveticus R0052* and *Bifidobacterium longum R0175* lifelong administration in rats fed with a high-fat diet attenuated the deleterious effects of the metabolic syndrome and reduced caloric intake in male but not female rats [8].

Indeed, the modulation of gut microbiota in obesity remains a challenge, and this is reviewed in the paper by Breton and co-authors [6]. The authors discuss this problem, highlighting the necessity of better understanding the molecular link between gut microbiota and the brain, as well as the recent development of new generation probiotics for diabetes and obesity treatment such as *Akkermansia muciniphila* and *Hafnia alvei*, the latter able to increase a feeling of satiety in overweight humans.

Specific *Lactobacillus* strain supplementation can be useful not only for the treatment of overweight and obesity but also in inverse metabolic situations, ex. during anorexia-induced by chemotherapy. As such, Marsova et al. in their study used a *Lactobacillus brevis* strain in mice treated with an anti-cancer drug 5-fluorouracil [9]. Although the authors did not report feeding behavior, they found an attenuation of gut inflammation in mice given the probiotic treatment, suggesting that this *Lactobacillus* strain may also diminish inflammation-induced anorexia. Such an effect is promising and should be confirmed in a further study, ideally in humans undergoing cancer chemotherapy.

While anorexia of chemotherapy may be due to dehydration secondary to gut inflammation and diarrhea, the origin of anorexia nervosa still remains unclear. Two papers within this Special Issue explore the role of gut microbiota in anorexia via two distinct molecular pathways involving cholecystokinin (CCK) and α-melanocyte-stimulating hormone (α-MSH) signaling, respectively. As such, in his conceptual paper, Stein Frostad proposed an original hypothesis that malnutrition and dysbiosis may increase the sensitivity to the satietogenic action of CCK, leading to a vicious circle of anorexia [10]. The other paper builds on the earlier study which identified *Escherichia coli* protein caseinolytic protease B (ClpB) as an antigen-mimetic of anorexigenic neuropeptide α-MSH [11]. In the present study, authors show that in rats, starvation and female sex, i.e., risk factors for developing anorexia nervosa, are both associated with an increased production of ClpB protein by gut microbiota [12]. These results contribute to the pathophysiological model of eating disorders [13].

Finally, in their review article, Thangaleela et al. discuss the first, rational experience of the use of probiotics in Parkinson’s disease (PD) [14]. Although altered feeding behavior in PD can be an independent symptom from the core problem of motor dysfunction experienced, such patients are treated with L-DOPA which may also influence the dopaminergic system regulating feeding behavior [15]. Currently, little attention is given to this aspect; however, it should be considered conjointly with anti-PD therapy.

In conclusion, all the papers presented in this Editorial bring new data and further confirm the role of gut microbiota in the maintenance of body homeostasis, including energy balance. Accordingly, pathological conditions accompanied by altered feeding behavior and energy balance have an impact on gut microbiota composition, the restoration and modification of which represent valid therapeutic targets.

## Data Availability

The information about the data availability supporting the reported results of cited articles is available in corresponding publications.
